# Performance of the Polydopamine-Graphene Oxide Composite Substrate in the Osteogenic Differentiation of Mouse Embryonic Stem Cells

**DOI:** 10.3390/ijms22147323

**Published:** 2021-07-07

**Authors:** Na Young Shim, Jung Sun Heo

**Affiliations:** Department of Maxillofacial Biomedical Engineering and Institute of Oral Biology, School of Dentistry, Kyung Hee University, 26 Kyunghee-daero, Dongdaemun-gu, Seoul 02447, Korea; sny4845@hanmail.net

**Keywords:** mouse embryonic stem cells, graphene oxide, polydopamine, osteogenic differentiation, integrins, bone morphogenetic receptors (BMPRs)

## Abstract

Graphene oxide (GO) is a biocompatible material considered a favorable stem cell culture substrate. In this study, GO was modified with polydopamine (PDA) to facilitate depositing GO onto a tissue culture polystyrene (PT) surface, and the osteogenic performance of the PDA/GO composite in pluripotent embryonic stem cells (ESCs) was investigated. The surface chemistry of the PDA/GO-coated PT surface was analyzed by scanning electron microscopy (SEM) and X-ray photoelectron spectroscopy (XPS). A high cell viability of ESCs cultured on the PDA/GO composite-coated surface was initially ensured. Then, the osteogenic differentiation of the ESCs in response to the PDA/GO substrate was assessed by alkaline phosphatase (ALP) activity, intracellular calcium levels, matrix mineralization assay, and evaluation of the mRNA and protein levels of osteogenic factors. The culture of ESCs on the PDA/GO substrate presented higher osteogenic potency than that on the uncoated control surface. ESCs cultured on the PDA/GO substrate expressed significantly higher levels of integrin α5 and β1, as well as bone morphogenetic protein receptor (BMPR) types I and II, compared with the control groups. The phosphorylation of extracellular signal-regulated kinase (ERK)1/2, p38, and c-Jun-N-terminal kinase (JNK) mitogen-activated protein kinases (MAPKs) was observed in ESCs culture on the PDA/GO substrate. Moreover, BMP signal transduction by SMAD1/5/8 phosphorylation was increased more in cells on PDA/GO than in the control. The nuclear translocation of SMAD1/5/8 in cells was also processed in response to the PDA/GO substrate. Blocking activation of the integrin α5/β1, MAPK, or SMAD signaling pathways downregulated the PDA/GO-induced osteogenic differentiation of ESCs. These results suggest that the PDA/GO composite stimulates the osteogenic differentiation of ESCs via the integrin α5/β1, MAPK, and BMPR/SMAD signaling pathways.

## 1. Introduction

Macro-, micro-, and nanoscale extracellular matrix (ECM) organization provides a dynamic microenvironment to facilitate pivotal cell functions, including cell survival, migration, proliferation, and differentiation [1,2]. The biophysical roles of the ECM include being a critical regulator of stem cell behavior and function. The developmental fate of embryonic stem cells (ESCs) is also determined not only by soluble signaling molecules but also by the ECM contents, comprising the stem cell niche [3]. Since many biomaterials for tissue engineering and regenerative medicine aim to modulate the ECM, which governs stem cell differentiation and tissue construction, identifying biological ECM substitutes and how the engineered ECM governs stem cell performance is critical.

Recently, carbon-based materials have received increased attention in various biomedical engineering applications, such as osteogenesis [4], bone replacement [5], and drug delivery systems [6]. Graphene, a carbon-based nanomaterial with versatile physicochemical properties, has attracted increased research attention in various bio-engineering fields [7]. Graphene is a flat monolayer of carbon atoms patterned into a honeycomb, two-dimensional (2D) lattice, and it serves as a biocompatible and implantable platform for stem cell culture and artificial microenvironments [8,9,10]. Moreover, the biofunctionalized capability of graphene and its derivative, graphene oxide (GO), has highlighted these carbon nanomaterials in regenerative medicine and biotechnology studies [11,12]. GO has been shown to strengthen induced pluripotent stem cell (iPSC) growth and to promote spontaneous differentiation [10]. Graphene and GO encouraged the cardiomyogenic or neuronal differentiation of ESCs [13,14]. GO-filmed substrates also intensified the differentiation of mouse ESCs to the hematopoietic lineage [15]. Growing evidence has indicated that a graphene surface provides a biocompatible nanoscale to accelerate the differentiation of human mesenchymal stem cells (hMSCs) into a bone cell specification without cellular toxicity [8,16,17]. GO-incorporated poly (lactic-co-glycolic acid) (PLGA) nanofiber structures also enhance the proliferation and osteogenic differentiation of hMSCs [18]. Collectively, these results indicate the potential of graphene and GO-based biomaterials for stem cell-related biomedical applications.

Graphene and its derivatives significantly promote ECM–integrin clustering signaling and promote the long-term adhesion of human neuronal stem cells [19,20]. GO also strengthens the adherence of mouse ESCs in controlling the ESC differentiation potency [21]. Moreover, the integrin signaling pathway is involved in the biocompatibility of GO for the self-renewal of ESCs [22]. Many integrin-connected signaling networks influence not only cell adhesion to ECM substrates but also specific cell lineage commitments [23,24,25]. Thus, cell-graphene/GO substrate interactions can contribute to considerable cellular processes from the onset of adhesion operation. 

Graphene and GO can be further functionalized by other biomaterials through various chemical bonds, such as covalent bonds, ionic bonds, and Van der Waals forces [26,27]. 

To establish effective strategies to fabricate functional matrixes or films, 3,4-dihydroxy-L-phenylalanine (DOPA) derived from mussel adhesive threads has been used because of its extraordinarily adhesion properties [28]. Dopamine (DA), a catecholamine that contains catechol and amine functional groups, can self-polymerize to polydopamine (PDA) at an alkaline pH level, coating any virtual surface and presenting mussel-derived adhesive properties [28]. Based on these properties, PDA can be applied as an intermediate layer to deposit GO onto various substrates. Thus, this study employed PDA to securely attach GO nanosheets to the surface and manufactured carbon-based nanoscale ECM with GO and PDA hybrid materials (PDA/GO).

Although several studies have reported the involvement of GO in ESC self-renewal and differentiation into the hematopoietic or neuronal lineage, to date, few studies have investigated the influences of GOs on the osteogenic differentiation of ESCs. The present study investigated how the PDA/GO composite substrate influences the osteogenic differentiation of ESCs. Moreover, the interaction of ESCs and this engineered microenvironment was demonstrated by certain signaling mechanisms to promote and control the fate of ESCs. 

## 2. Results

### 2.1. PDA/GO Substrate Promotes the Osteogenic Differentiation of ESCs

**Figure 1:**
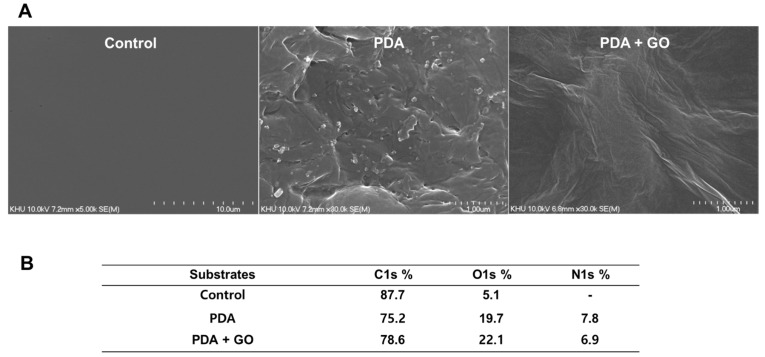
Surface characterization of PDA/GO substrates. (**A**) Topographical features of the PT surface, PDA (1 mg/mL)-coated, and PDA/GO (1 mg/mL + 1 mg/mL)-coated surfaces were assessed by scanning electron microscopy at ×10 k magnification. (**B**) Relative atomic composition of each sample.

The morphology of the patterned PDA/GO substrate was first observed by SEM. The SEM images demonstrated a relative PDA coating or multilayered PDA/GO layer on the substrate (Figure 1A). The atomic constitution by XPS indicated that C1s is the most predominant element on the PT surface. The PDA-coated surface demonstrated increased concentrations of O1s and N1s compared with that on the PT surface. When PDA/GO was deposited on the surfaces, the concentrations of C1s and O1s were increased compared with those on the PDA surface (Figure 1B). These results suggest that the PDA or PDA/GO substrate was successfully immobilized on the PT culture surface. 

**Figure 2:**
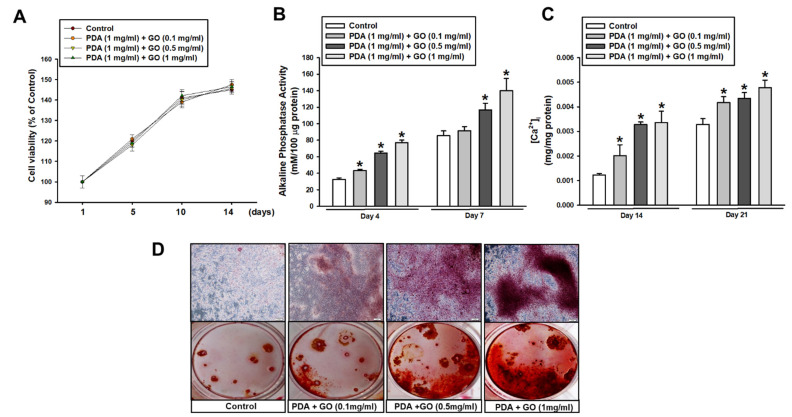
Osteogenic differentiation of ESCs cultured on PDA/GO-modified surfaces. (**A**) Cells were cultured on the PDA/GO substrate (composite of PDA with 1 mg/mL and variable GO concentration with 0.1, 0.5, or 1 mg/mL) for 1, 5, 10, and 14 days, and then, the cell viability was assessed as described in the Materials and Methods. (**B**) ALP activity, (**C**) [Ca^2+^]_i_, and (**D**) Alizarin Red S staining were evaluated after 4, 7, 14, or 21 days of osteogenic induction. The values are presented as means ± SD (*n* = 3). * *p* < 0.05 vs. the control value at each time point.

**Figure 3:**
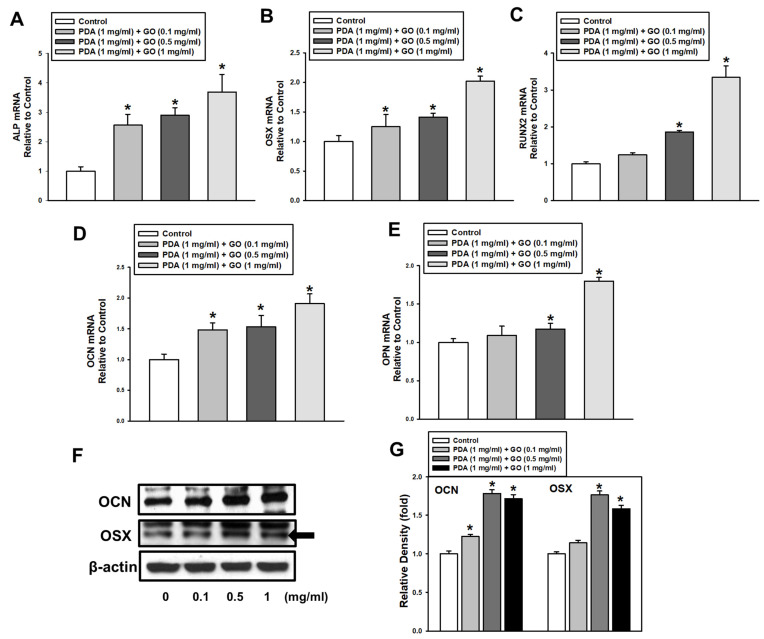
Effect of PDA/GO on osteogenic-related gene and protein expression. (**A**–**E**) The mRNA expression of ALP, OSX, RUNX2, OCN, and OPN was analyzed after 7-day osteogenic induction by real-time RT-PCR. (**F**) The protein levels of OCN (5.5 kDa) and OSX (45 kDa) were determined by Western blot. (**G**) The bars denote the density relative to β-actin. The values are expressed as means ± SD (*n* = 3). * *p* < 0.05 vs. control value.

Before observing the osteogenic response of ESCs, cell viability on PDA/GO was first assessed after cells were cultured on PDA/GO-coated (0.1, 0.5, 1 mg/mL) or uncoated (control) surfaces for 1, 5, 10, and 14 days. No difference was found in the cell viability between ESCs cultured on the PDA/GO and control surface, indicating no cytotoxicity of the present combination of PDA/GO substrates (Figure 2A). Subsequently, the osteogenic differentiation of ESCs was explored by analyzing the ALP activity and intracellular calcium levels ([Ca^2+^]_i_). The ALP activity in cells on the PDA/GO substrate was increased compared with those of the control group on day 4 and was further increased on day 7 (Figure 2B). Similar to ALP activity, the [Ca^2+^]_i_ was also increased in cells on PDA/GO in a dose-dependent manner (Figure 2C). Extracellular calcium deposits were also analyzed. Figure 2D indicates that calcium deposits were increased in ESCs on the PDA/GO substrate compared with those of the control groups (Figure 2D). The osteogenic effect of the PDA/GO substrate was assessed by following the gene and protein expression of the osteogenic markers. The mRNA expression of osteogenic target genes (ALP, osterix (OSX), runt-related transcription factor 2 (RUNX2), osteocalcin (OCN), and osteopontin (OPN)) was increased in cells on a PDA/GO coating compared with that of the control group and showed the highest level in cells on 1 mg/mL of PDA/GO (Figure 3A–E). Western blot analysis also showed that OCN and OSX protein levels were increased in cells cultured on PDA/GO substrate on day 7 of osteogenic induction (Figure 3F,G).

### 2.2. Integrin α5/β1 and BMPR I/II Signaling Pathways in ESCs on the PDA/GO Substrate 

**Figure 4:**
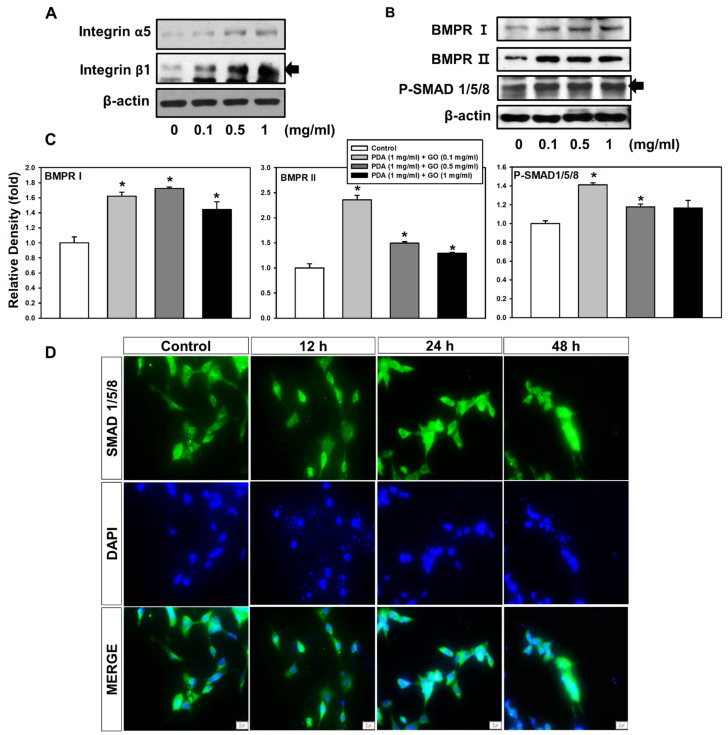
Effect of PDA/GO on integrin α5/β1, MAPKs, BMPR I/II, and SMAD 1/5/8 signaling pathways. The cells were cultured on the PDA/GO substrate for 7 days, and the protein levels of (**A**) integrin α5 (138 kDa), integrin β1 (150 kDa), and (**B**) BMPR I (50–55 kDa), BMPR II (115 kDa), and phosphorylation levels of SMAD 1/5/8 (52–56 kDa) were analyzed. (**C**) The bars denote the density relative to β-actin. The values are expressed as means ± SD (*n* = 3). * *p* < 0.05 vs. control value. (**D**) Nuclear translocation of SMAD 1/5/8 was assessed by immunofluorescence staining (scale bar, 20 μm).

To understand the molecular mechanisms underlying the link between ESCs and the PDA/GO substrate, we explored whether integrins, as an adhesion receptor, and bone morphogenetic protein receptors (BMPRs), as representative osteogenic-functioning receptors, are associated with PDA/GO substrate-derived ESC-osteolineage commitment. The protein levels of integrins were analyzed in cells cultured on the PDA/GO substrate after 7 days of culture (Figure 4A). The α5 and β1 subunits of integrins showed increased protein levels in cells on the PDA/GO substrate compared with those in the control groups (Figure 4A). These increases were dose dependent according to the GO concentration. Western blot analysis showed that the type I and II BMPR levels were significantly increased when cells were cultured on the PDA/GO substrate (Figure 4B). SMAD 1/5/8 are signal transducers, which are activated by BMP receptors and mediate BMP signaling pathways. SMAD1/5/8 phosphorylation was elevated in cells on the PDA/GO substrate than in the control (Figure 4B,C). The nuclear translocation of SMAD1/5/8 was also confirmed using immunofluorescence staining (Figure 4D). Thus, activation of the BMPR-SMAD1/5/8 signaling pathway may provide an additional osteoinductive signal for ESC-osteogenic differentiation on the PDA/GO substrate.

**Figure 5:**
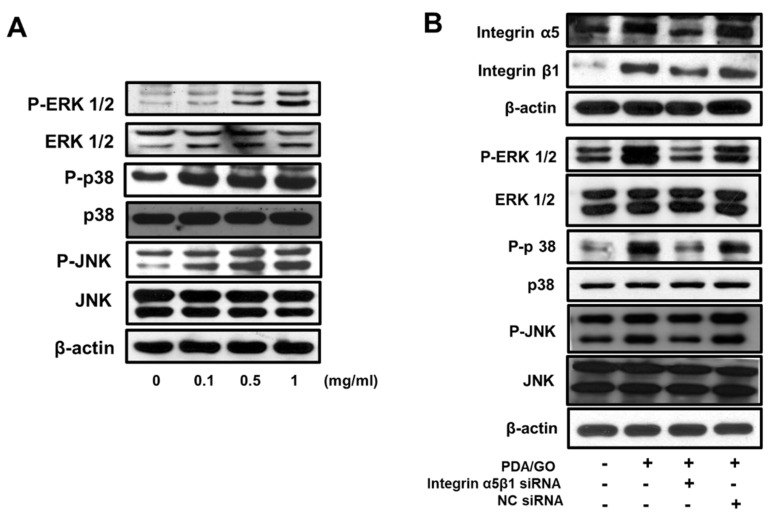
Effect of PDA/GO on MAPKs signaling pathways. The cells were cultured on the PDA/GO substrate for 7 days and (**A**) Phosphorylation of ERK1/2 (42–44 kDa), p38 (38 kDa), and JNK (46–54 kDa) was assessed. (**B**) Cells were transfected with integrin α5/β1-specific siRNA, and the protein levels of integrin α5, β1, and the ERK1/2, p38, and JNK phosphorylation levels were examined after 4 days of osteogenic induction.

This study also examined whether ESC osteogenic differentiation on the PDA/GO substrate requires the activation of mitogen-activated protein kinases (MAPKs), which are responsible for osteogenic differentiation and bone formation [29,30]. The activation of MAPKs, such as extracellular signal-regulated kinase (ERK)1/2, p38, and c-Jun-N-terminal kinase (JNK), was assessed by measuring the phosphorylated form of each MAPK using Western blot analysis (Figure 5A). The expression of P-ERK1/2, P-p38, and P-JNK was upregulated in cells on the PDA/GO substrate compared with that in the control. No significant differences were found in the expression of total ERK1/2, p38, and JNK among all the experimental groups. To further understand this extracellular-leading intracellular signaling pathway, the influence of integrin α5/β1 in MAPK activation was evaluated using integrin α5/β1 siRNA. The knockdown efficiency of integrin α5/β1 siRNA was first confirmed when the transfection of integrin α5/β1 siRNA downregulated the protein levels of each integrin in cells on the PDA/GO substrate (Figure 5B). The phosphorylation of ERK1/2, p38, and JNK was decreased by the knockdown of integrin α5/β1, indicating that integrin α5/β1 mediates MAPK signaling pathways during ESC osteogenic differentiation in response to the PDA/GO substrate.

### 2.3. Integrin α5/β1, MAPKs, and BMPRs/SMAD Mediate ESC Osteogenic Differentiation on the PDA/GO Substrate

**Figure 6:**
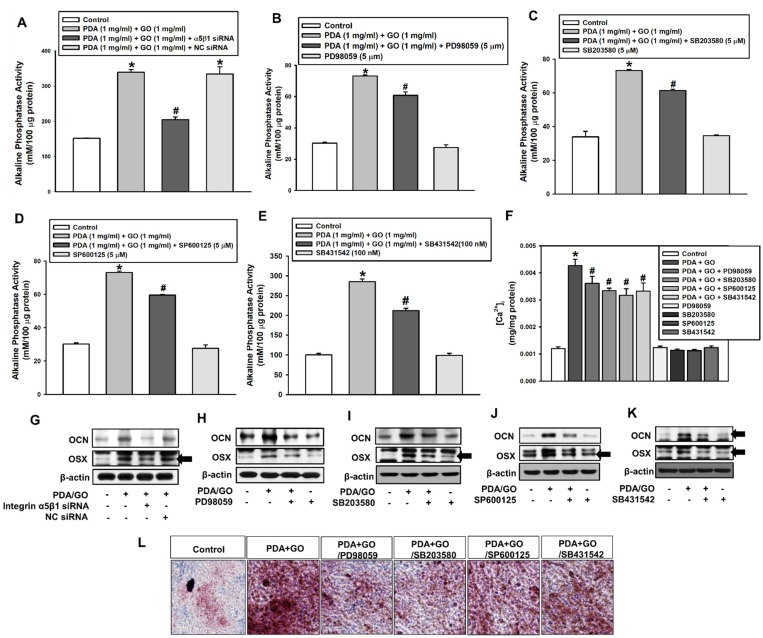
Role of the integrin α5/β1, MAPK, and BMPR/SMAD signaling pathways in the PDA/GO-mediated osteogenic differentiation of ESCs. Cells were transfected with integrin α5/β1-specific siRNA, or treated with PD98059 (5 μM), SB203580 (5 μM), SP600125 (5 μM), or SB431542 (100 nM) for 48 h before (**A**–**E**) ALP activity, (**F**) [Ca^2+^]_i_, (**G**–**K**) Western blot analysis, and (**L**) Alizarin Red S staining. The values are expressed as means ± SD (*n* = 4). * *p* < 0.05 vs. each control value, and ^#^ *p* < 0.05 vs. PDA/GO value.

Considering that the activation of integrin α5/β1, MAPK, and BMPR/SMAD signaling pathways is related to PDA/GO-derived ESC osteogenesis, we investigated the influence of individual pathways in the osteogenic differentiation of ESCs on the PDA/GO substrate. Figure 6A demonstrates that the knockdown of integrin α5/β1 reduced the PDA/GO-induced ESC ALP activity. Western blot analysis confirmed the downregulation of OCN and OSX protein levels after integrin α5/β1-knockdown (Figure 6G). Next, the connection between MAPK activation and PDA/GO-derived ESC osteogenesis was examined. When cells were treated with ERK inhibitor (PD98059), p38 inhibitor (SB203580), JNK inhibitor (SP600125), or SMAD inhibitor (SB431542), the ALP activity and [Ca^2+^]_i_ of ESCs cultured on the PDA/GO were downregulated (Figure 6B–F), as well as OCN and OSX protein levels (Figure 6H–K). Calcium deposits were also decreased in ESCs on the PDA/GO substrate with each inhibitor (Figure 6L).

## 3. Discussion

The present study showed that a PDA/GO composite-coated cell culture substrate can effectively contribute to ESC osteogenic differentiation through the integrin α5/β1, MAPK, and BMPR/SMAD signaling pathways. There is an increased demand for optimized microenvironmental systems to maintain self-renewal or facilitate the differentiation of adult and embryonic stem cells [31,32]. Previous studies have shown that novel biomaterials that imitate the extracellular microenvironment and in vivo construction encourage the efficient differentiation of stem cells to the desired cell lineage [33,34]. Thus, the present study demonstrated that the culture of ESCs on the PDA/GO-coated surface promotes osteogenic differentiation of ESCs. Few studies have investigated the osteogenic effect of the PDA/GO composite on ESCs and even on MSCs. One study showed that a PDA-inspired GO and titanium scaffold promoted bone marrow-derived MSC adhesion and proliferation, as well as development of nanostructured environments for bone regeneration [35]. Our PDA/GO-functionalized culture substrate is a potential strategy to produce large numbers of osteogenic cells from ESCs. 

The interplay between stem cells and the engineered extracellular microenvironment for a practical stem cell differentiation system must be investigated further. The present study provides the underlying molecular processes for ESC osteogenic differentiation on a physicochemical PDA/GO substrate. The first cue mediating the mechanical signal of PDA/GO to ESCs was the cell surface receptors integrin α5/β1 in the present study. Previous studies have shown that the gene and protein expression levels of integrin α5/β1 were increased during human MSC differentiation to osteoblasts [36,37,38]. Osteoblast adhesion on certain ECM proteins was achieved through binding to αvβ1 integrin [39]. Various studies have demonstrated that dynamic expression of different integrins is required for the osteogenic differentiation of MSCs [40,41]. The use of graphene material suggests the pivotal role of integrin β1 in ECM roughness recognition, which is involved in osteoblast maturation and MSC differentiation on graphitic carbon-coated surfaces [42]. Other reports have shown that the protein expression of integrin β1 is increased on graphene-coated Si/SiO_2_ substrates by significantly promoting the differentiation of MSCs into bone cells [8]. Integrin β1 binds 12 different α subunits, including the α5 subunit, in osteoblasts and osteoprogenitor cells. Moreover, integrin β1 mediates cell adhesion to bone matrix and promotes osteogenic cell proliferation and differentiation, indicating that integrin β1 signaling plays a major function in bone formation [43,44]. To date, numerous studies have shown that integrins α5/β1 participate in the osteogenic differentiation of osteoprogenitors and MSCs as previously mentioned. We also suggest that integrin α5/β1 can introduce the differentiation of ESCs into the osteogenic lineage when they are cultured on the PDA/GO substrate.

In the present study, integrin α5/β1 led to the activation of ERK1/2, p38, and JNK MAPKs as outside-relayed intracellular pathways during ESC osteogenic differentiation in response to PDA/GO. These MAPKs are the best-characterized downstream signaling pathways of the matrix microenvironment–integrin interactions in osteogenic cell types [44,45,46]. Consistent with the current results, MAPKs have been frequently reported as a key player for the osteogenic differentiation of various types of stem cells [47,48,49]. Regarding previous studies and our findings, these integrin–MAPK stepwise processes trigger the osteogenic induction of ESCs in response to the PDA/GO culture substrate.

Interestingly, the BMP receptors, members of the transforming growth factor-β (TGF-β) superfamily, were suggested as the other cell-receiving signals from the PDA/GO substrate. GO mechanistically interacts with multiple cell surface receptors [50,51,52]. However, the PDA or GO material can activate BMP receptors in ESCs or other stem cell models. One previous study reported that GO activated TGF-β receptor/SMAD2/3 signaling to trigger new metastases of human cancer cells [53]. Insufficient data exist to identify PDA/GO-related osteogenic signaling pathways; however, our findings showed that the PDA/GO substrate facilitates the presentation of BMP receptors in ESCs and enhances ESC osteogenic activity. BMPRs and canonical SMAD signaling are widely studied in the bone biology field [54,55,56]. Several studies have verified that SMAD-dependent BMP and TGF-β signaling pathways manage both osteoblast and osteoclast function; thus, they play potential roles in skeletal development, bone formation, and bone homeostasis [57,58,59,60]. The present study demonstrated increased BMPR type I and II protein levels and activation of SMAD 1/5/8, receptor-regulated SMADs (R-SMADs), which are responsible for PDA/GO-derived ESC differentiation into osteolineage cells. A previous study reported that BMPR recognition and the phosphorylation of SMAD 1/5/8 signaling promoted the in vitro osteogenic differentiation of C2C12 cells in a magnesium-modified calcium phosphate matrix model [61]. Thus, the designed ECM substrates sensing the BMPR/SMAD signaling axis enable biomaterials to attend osteoinductive performance.

Although the present study suggests a model for ESC osteogenic differentiation on PDA/GO composite by the initiation of both integrin α5/β1 and BMPRs, the reciprocal interactions between these receptors is still unclear. Further study of this ambiguous issue is warranted. 

**Figure 7:**
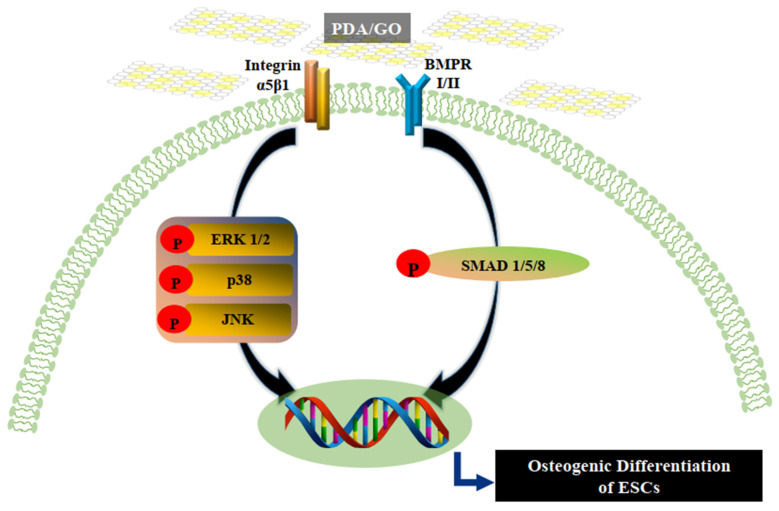
Hypothesized model of the PDA/GO-mediated osteogenic differentiation of ESCs. When ESCs are cultured on PDA/GO-modified surfaces, integrin α5/β1 and BMPR I/II are recognized, leading to MAPK or SMAD signaling pathway activation and eventually promoting the osteogenic differentiation of ESCs.

This study systematically investigated the osteogenic bioactivity of PDA/GO composite as a substrate material with ESCs. When ESCs were cultured on PDA/GO substrates, cells significantly exhibited the osteogenic differentiation through integrin α5/β1, MAPK, and BMPR I/II-SMAD 1/5/8 signaling pathways (Figure 7). Finally, the PDA/GO culture system may provide a stem cell niche–mimetic environment to control stem cell fate and a facile and promising strategy for bone tissue engineering and regenerative medicine. 

## 4. Materials and Methods

### 4.1. Materials

Fetal bovine serum (FBS) was supplied by Gibco-BRL (Gaithersburg, MD, USA). Antibodies used for Western blot analysis and immunofluorescence staining were obtained from Santa Cruz Biotechnology (Santa Cruz, CA, USA). The chemicals, including L-3,4-dihydroxyphenylalanine (L-DOPA) and graphene oxide dispersion, were purchased from Sigma Chemical Company (St. Louis, MO, USA). Other laboratory materials were acquired from SPL Lifescience (Pocheon, Korea).

### 4.2. Mouse ESC Culture and Embryoid Body Formation

Mouse ESCs (ES-E14TG2a (ATCC^®^ CRL-1821™)) were supplied from the American Type Culture Collection (Manassas, VA, USA). ESC culture was performed as in our previous report [62]. To develop embryoid bodies (EBs), dissociated cells were aggregated by hanging drop with 2000 cells in 20 μL of DMEM. We used 5-day-old EBs in every experiment.

### 4.3. Preparation of the PDA/GO Composite Substrate

The PDA solution was prepared by dissolving 1 mg of L-DOPA in 1 mL of 10 mM Tris buffer base (pH 8.5; Sigma-Aldrich, St. Louis, MI, USA). Then, GO dispersion was added to the PDA solution (1 mg/mL) under magnetic stirring at room temperature for 24 h. The final concentration of GO in the PDA solution was 0.1, 0.5, or 1 mg/mL for individual experiments. The polystyrene (PT) culture surface was coated with PDA/GO composite solution overnight at room temperature and washed three times with sterile phosphate-buffered saline (PBS). Then, the PDA/GO-modified surfaces were dried in a vacuum oven. 

### 4.4. Characterization of the PDA/GO-Coated Surface

Images of PDA/GO composite-coated surfaces were identified with scanning electron microscopy (SEM; S-4700, Hitachi, Tokyo, Japan). The specimens were rinsed with PBS and then freeze-dried overnight before SEM operation. The atomic composition of the PDA/GO-modified surfaces was assessed by X-ray photoelectron spectroscopy (XPS) as in our previous report [63]. The surface composition amount was obtained from the XPS survey spectra.

### 4.5. Cell Viability Assay

Cell viability was performed using the cell counting kit-8 (CCK-8) assay as in our previous report [62]. Briefly, cells were cultured on PDA/GO substrate (0, 0.1, 0.5, 1 mg/mL) for 1, 5, 10, and 14 days, and the CCK-8 assay was performed according to the manufacturer’s instructions. The optical density was observed at a wavelength of 450 nm using an ELISA reader system (Triad; DYNEX, Chantilly, VA, USA). The proportion of cell viability was designated relative to the control. 

### 4.6. Alkaline Phosphatase Activity Assay

Alkaline phosphatase activity was assessed as in our previous report [62]. Briefly, cells were plated onto PDA/GO composite-coated 60 mm dishes (10–15 EBs per dish). ALP activity was evaluated after 4 and 7 days of osteogenic induction using the *p*-nitrophenylphosphate (pNPP) procedure. The ALP enzyme activity was denoted as mM/100 μg of protein.

### 4.7. Intracellular Calcium Quantification Assay

Quantification of the intracellular calcium level was evaluated based on our previous report [62]. Briefly, cells were plated onto PDA/GO composite-coated 60 mm dishes (10–15 EBs per dish). Fourteen and twenty-one days after osteogenic induction, the intracellular calcium concentration was measured using a calcium assay kit (BioAssay Systems, Hayward, CA, USA) according to the manufacturer’s information. The optical density was read at 612 nm. The calcium level was expressed as mg/mg of protein.

### 4.8. Alizarin Red Staining 

Alizarin Red staining was achieved as described in our previous report [64]. The cells were fixed with 4% paraformaldehyde for 15 min and rinsed three times with PBS. Then, the cells were stained with 2% Alizarin Red S solution (pH 4.2) for 5 min, and unbound dye residue was washed with PBS. The stained images of different surfaces were observed using a light microscope. 

### 4.9. Osteogenic-Related Gene Expression Analysis

Real-time reverse transcription-polymerase chain reaction (RT-PCR) was conducted as in our previous report [62] to measure the mRNA expression of osteogenic genes. The primers used were as follows: 5′-GAC TGG TAC TCG GAT AAC GA-3′ (forward) and 5′-TGC GGT TCC AGA CAT AGT GG-3′ (reverse) for ALP; 5′-CCAACTTCCTGTGCTCCGTG-3′ (forward) and 5′-TCTTGCCTCGTCCGCTCC-3′ (reverse) for Runx2; 5′-TGA AAC GAG TCA GCT CTG GAT G-3′ (forward) and 5′-TGA AAT TCA TGG CTG TGG AA-3′ (reverse) for OPN; 5′-TGA GGA GGA AGT TCA CTA TGG-3′ (forward) and 5′-TTC TTT GTG CCT GCT TTG C-3′ (reverse) for OSX; 5′-ATG AGA GCC CTC ACA CTC CTC-3′ (forward) and 5′-GCC GTA GAA GCG CCG ATA GGC-3′ (reverse) for OCN; and 5′-GCT CTC CAG AAC ATC ATC C-3′ (forward) and 5′-TGC TTC ACC ACC TTC TTG-3′ (reverse) for GAPDH.

### 4.10. Western Blot Analysis

Western blot analysis was performed as in our previous report [63]. Briefly, the primary (anti-OCN, anti-OSX, anti-integrin α5, anti-integrin β1, anti-BMPR I, anti-BMPR II, anti-SMAD1/5/8, anti-P-SMAD1/5/8, anti-ERK1/2, anti-P-ERK1/2, anti-p38, anti-P-p38, anti-JNK, anti-P-JNK, or anti-β-actin; Santa Cruz Biotechnology) and secondary antibodies (goat anti-rabbit immunoglobulin G (IgG) or goat anti-mouse IgG conjugated to horseradish peroxidase) were employed with the dilutions recommended by the supplier. The blots were developed using enhanced chemiluminescence (Santa Cruz Biotechnology) and developed using X-ray film (Eastman-Kodak, Rochester, NY, USA). 

### 4.11. Immunofluorescence Staining 

Nuclear translocation of SMAD1/5/8 was detected by time-dependent immunofluorescence staining. The cells were incubated with SMAD1/5/8 antibodies at 4 °C overnight and then with Alexa Fluor 488 goat anti-rabbit IgG for 2 h. Fluorescence images were developed using a fluorescence microscope (Fluoview 300; Olympus, Tokyo, Japan).

### 4.12. SiRNA Transfection

Small interfering RNA (siRNA) transfection was conducted as in our previous report [64]. Briefly, the cells were transfected with either an integrin α5/β1 siRNA (25 nM) or a negative control siRNA (scrambled) for 48 h using a transfection reagent (RNAiMAX, Invitrogen, Waltham, MA, USA) according to the manufacturer’s manual before being subjected to ALP activity and Western blot analysis.

### 4.13. Statistical Analysis

All the data were expressed as means ± standard deviation. One-way analysis of variance was used for multiple comparisons (Duncan’s multiple range test). Analyses were obtained using SPSS software (ver. 10.0; SPSS Inc., Chicago, IL, USA). A *p*-value < 0.05 were considered statistically significant.

## Data Availability

Not applicable.
